# Interpenetration of Natural Polymer Aerogels by Supercritical Drying

**DOI:** 10.3390/polym8040106

**Published:** 2016-03-24

**Authors:** Lucia Baldino, Simona Concilio, Stefano Cardea, Ernesto Reverchon

**Affiliations:** Department of Industrial Engineering, University of Salerno, Via Giovanni Paolo II, 132, 84084 Fisciano (SA), Italy; lbaldino@unisa.it (L.B.); ereverchon@unisa.it (E.R.)

**Keywords:** alginate, gelatin, aerogel, biomaterials, supercritical drying

## Abstract

Natural polymers, such as alginate and gelatin, can be used to produce scaffolds for tissue engineering applications; but, their mechanical and biochemical performance should be improved. A possible solution to obtain this result, is the generation of multi-component scaffolds, by blending two or more polymers. One way to realize it, is the formation of an interpenetrating polymer network (IPN). In this work, the interpenetration of alginate and gelatin hydrogels has been successfully obtained and preserved by supercritical CO_2_ (SC-CO_2_) drying performed at 200 bar and 35 °C, using different blend compositions: from alginate/gelatin = 20:80 *v*/*v* to alginate/gelatin = 80:20 *v*/*v*. The process allowed modulation of morphology and mechanical properties of these blends. The overall result was made possible by the supercritical drying process that, working at zero surface tension, allows preserving the hydrogels nanostructure in the corresponding aerogels.

## 1. Introduction

Tissue-engineering (TE) involves three major components: (1) cells, (2) scaffold, and (3) tissue formation environment. Scaffold should mime the natural features of the tissue extracellular matrix (ECM) at macro, micro, and nanoscale and should provide an initial biomechanical profile for the replaced tissue, allowing cells to develop their functions in a simulated environment as they would *in vivo* [1]. The most complex characteristic that should be embedded in the scaffold is nanoscale feature due to the difficulty in manipulating the matter at nanoscale level.

Scaffolds can be obtained from natural or synthetic polymers. Natural polymers, such as collagen [2,3], gelatin [4,5], fibrin [6,7], chitosan [8,9], and alginate [10,11], have gained large interest for TE applications, due to their biocompatibility, capacity to adsorb large quantities of water, and ability to assume morphologies similar to the ECM and biodegradability [12,13,14].

Alginate and gelatin are among the most used natural polymers in TE applications. Alginate is a negatively charged linear polysaccharide composed of 1,4-linked β-d-mannuronate (M) and 1,4-linked α-l-guluronate (G) residues. G-blocks of alginate can generate an “egg box”-like structure hydrogel in contact with divalent cations, such as Ca^2+^, Ba^2+^, and Sr^2+^ [15]. It is largely used in the biomedical field, due to its biodegradability, biocompatibility, hydrophilicity, and low toxicity [16]. Nevertheless, its negative charge inhibits protein adsorption and reduces cellular adhesion. For this reason, bioactive molecules such as arginine-glycine-aspartic acid (RGD) and fibronectin were proposed for the immobilization within the hydrogel, to induce cell adhesion [17,18].

Gelatin is formed by denatured collagen; it has relatively low antigenicity compared to its precursor and maintains signals that may promote cell adhesion, differentiation, and proliferation, such as the RGD sequence of collagen [19]. It is largely soluble in aqueous solutions; therefore, it is generally crosslinked with glutaraldehyde (GTA) to increase both its thermal and mechanical stability [20,21]; but, GTA is cytotoxic and it may be desirable to minimize its use or to try to reduce its unreacted residues after processing [22].

Alginate and gelatin are frequently prepared in the form of hydrogels, since gels can reproduce a nanostructured fibrous network, similar to native ECM and have been proposed in soft tissue applications, or dried by lyophilization, for hard tissues application. In this last case, the scaffold can collapse, due to the surface tension exerted by the solvent on the polymer matrix during the drying process. Moreover, they are generally characterized by poor mechanical properties and could not have the biological active groups required to interact with cells. Cheng *et al.* prepared alginate-based aerogels by ionotropic gelation and freeze drying, using *N*,*N*′-methylenebisacrylamide and carboxy-methylcellulose as reinforcing agents. The final aerogels showed an irregular and closed morphology [23]. Yamamoto *et al.* obtained alginate scaffolds with aligned micropores by freeze drying [24]. Wu *et al.* prepared aligned porous scaffolds of gelatin by unidirectional freeze-drying method [4]. Zhang *et al.* produced 3-D macroporous gelatin/hyaluronic acid hybrid scaffolds using the same technique [25].

A possible solution to improve mechanical and biochemical performance of alginate and gelatin is the generation of multi-component scaffolds, obtained by blending the two polymers. One way to realize it is the formation of an interpenetrating polymer network (IPN) between alginate and gelatin. In particular, a non-covalent IPN could be realized, in which gelatin and alginate are crosslinked and partially interlocked on a molecular scale, but not covalently bonded to each other [26]. It can give the opportunity of modulating architectural, mechanical, and biological properties of the blends. However, the production of polymer blends can, in principle, be complex due to compatibility problems among the polymeric chains that could give, for example, phase separation during the blend formation [27,28].

To improve alginate mechanical resistance, some authors prepared alginate blends with other polymers [29,30] or inorganic substances [31,32]. Dahlmann *et al.* developed an hydrogelation system, based on alginate and hyaluronic acid, in which aldehyde and hydrazide-derivatives enabled covalent hydrazone cross-linking of polysaccharides in the presence of myocytes [33]. Liu *et al.* produced hydrogels based on dextran modified with methacrylate and aldehyde groups mixed with gelatin, using freeze-drying. The incorporation of gelatin into the hydrogels provided cell adhesive and enzymatically degradable properties and significantly increased the compressive modulus and strength of the polymeric system [34]. Gautam *et al.* used electrospinning to prepare composite nanofibrous tissue engineering-scaffolds consisting of polycaprolactone and gelatin. They demonstrated cell adhesion to the composite scaffold and the expression of the characteristic cell morphologies indicated the suitability of the scaffold for TE applications [35]. Rosellini *et al.* blended alginate and gelatin, to obtain films for myocardial tissue engineering, produced by solvent evaporation. They showed the presence of interactions among the functional groups of the two biopolymers [36]. However, until now, morphological analyses only in rare cases have been performed and highlighted the absence of an organization at nanoscale of the polymeric construct, probably due to the processes used for sample production.

One of the techniques used for biopolymer aerogel production is freeze drying. Various studies on different polymer systems have been performed using this technique; however, the authors generally obtained microporous aerogels that did not exhibit a sub-nanostructure. It is well known, instead, that this last feature is relevant for cell adhesion on the artificial support in tissue engineering applications. The scientific literature contains several works on alginate and gelatin scaffolds produced by freeze drying. However, also in these cases, aerogels characterized only by a microporous morphology have been obtained [4,23,24,25]. Generally speaking, freeze drying leads to formation of microporous structures and pore size mainly depending on freezing temperature, solute concentration, and kind of solvent. In particular, when an aqueous solution is frozen at an extremely low temperature (−196 °C) the rapid formation of ice nuclei leads to a growth of small ice crystals; but, in any case, not of nanometric dimension [37].

Processes assisted by supercritical CO_2_ (SC-CO_2_) have been proposed as alternative to traditional ones, for example for micro and nanoparticle production [38,39,40,41], extraction [42], and membrane preparation [43,44]. In particular, supercritical gel drying has been demonstrated to be an efficient technique to obtain aerogels that maintain their native morphology at micro and nanoscale, and present very small organic solvent residues in the final structure, making it safe for biomedical applications [22,45]. For example, García-González *et al.* reviewed polysaccharide aerogel production by SC-CO_2_ for biomedical and pharmaceutical applications [46], whereas Mikkonen *et al.* described polysaccharide aerogels as advanced food materials [47]. According to Ulker *et al.*, both inorganic and organic aerogels present a huge potential in the field of drug delivery. In particular, depending on the structural properties, the adsorption or release of the active compounds can be obtained by tailoring the synthesis conditions [48].

These results are possible, because these processes are carried out at negligible surface tension and the supercritical mixture (solvent + CO_2_) shows a large mass transfer coefficient. These characteristics, in the case of gels, avoid structure collapse and the solvent is rapidly extracted from the polymeric matrix [49].

Therefore, the aim of this work is to produce alginate/gelatin (A/G) IPN aerogels by supercritical gel drying to evaluate if, using this technique, it is possible to obtain a good interpenetration of the two polymers at nanometric level, to preserve gels nanostructure and to analyze the mechanical performance of the blend. The obtained structures have been characterized by Field Emission Scanning Electron Microscopy (FESEM), Differential Scanning Calorimetry (DSC), Energy Dispersive X-ray spectroscopy (EDX). UV/Vis spectrophotometry has been used to measure GTA residues. Mechanical tests on A/G aerogels have also been performed.

## 2. Materials and Methods

PROTANAL LF 10/60 was termed high-G alginate, with M/G = 25/75 and was kindly provided by FMC BioPolymer (Milano, Italy); gelatin, type B from bovine skin, Calcium Chloride, Glutaraldehyde solution 25% *w*/*w* in water and Ethanol (purity > 99.8%) were purchased from Sigma-Aldrich (Milano, Italy); Carbon Dioxide (99% purity) was bought from Morlando Group S.R.L. (Sant’Antimo (NA), Italy). Distilled water was produced using a laboratory water distiller supplied by ISECO (St. Marcel (AO), Italy). All materials were used as received.

### 2.1. Preparation of Alginate/Gelatin Aerogels

#### 2.1.1. Experimental Procedure to Produce Alginate Aerogel

To obtain an alginate hydrogel, solutions of alginate in water, at 5% *w*/*w* concentration, were prepared and stirred for 24 h at 200 rpm. In particular, 0.5 g of alginate (Protanal LF 10/60) were dissolved in 10.0 mL of distilled water. Then, samples were produced by pouring the solution into cylindrical molds of 2 cm diameter and thickness of about 2 mm.

The samples were immersed in a 50 mL coagulation bath of CaCl_2_ (2.63 g, 5% in water) for 24 h to promote gelation. During this part of the process, sodium alginate was converted to calcium alginate. Then the hydrogels were removed from the molds and repeatedly washed with distilled water to eliminate Ca^2+^ residues. Subsequently, they were treated using a multistage solvent exchange in baths containing water and ethanol, with increasing concentrations of ethanol (10, 30, 50, 70, 90, and 100% *v*/*v*), 30 min each. Indeed, a water elimination step was required to perform the supercritical process, since CO_2_ and water, at the usual operative conditions, show a very reduced affinity [50].

#### 2.1.2. Experimental Procedure to Produce Gelatin Aerogel

A gelatin (Type B) solution 5% *w*/*w* in distilled water was prepared by dissolving 0.5 g of gelatin in 10.0 mL of water. The solution was stirred for 24 h at 200 rpm. Then, we produced samples by pouring the solution into cylindrical molds of 2 cm diameter and thickness of about 2 mm. The samples were immersed in a coagulation bath of 30 mL GTA (aqueous GTA, 25% *w*/*w*) 8% *v*/*v* in water, for 24 h in the dark, to obtain gelatin crosslinking and gelation. The obtained hydrogels were, then removed from the molds and repeatedly rinsed with distilled water to remove GTA residues. Then, these hydrogels also underwent a process of multistage solvent exchange in baths of water and ethanol with increasing concentrations of ethanol (10, 30, 50, 70, 90, and 100% *v*/*v*), 30 min each, progressively eliminating water.

#### 2.1.3. Experimental Procedure to Produce Alginate/Gelatin Aerogel

Two aqueous solutions of alginate (5% *w*/*w*) and gelatin (5% *w*/*w*) were prepared, as described before. The solutions were stirred for 24 h at 200 rpm. Then, the solutions of alginate and gelatin were mixed in three different ratios by volume: A/G—20/80, 50/50, 80/20. The mixtures were stirred for 1 h and, then, poured into cylindrical molds of 2 cm diameter and thickness of about 2 mm. Each sample was immersed in 25 mL of CaCl_2_ at 5% *w*/*w* in water for 24 h, to promote Alginate gel formation. The obtained hydrogels were, then, repeatedly washed with distilled water to remove Ca^2+^ residues. Afterwards, samples were immersed in 30 mL of an aqueous solution of GTA (aqueous GTA, 25% *w*/*w*) 8% *v*/*v* to induce the crosslinking of gelatin. A/G hydrogels were rinsed three times in distilled water to remove excess GTA and underwent to the same process of multistage exchange of the solvent used for single polymer hydrogels.

### 2.2. Supercritical Gel Drying

A, G and A/G aerogels were prepared using a homemade laboratory plant that consists of a 316 stainless steel cylindrical high-pressure vessel with an internal volume of 200 mL, equipped with a high pressure pump (mod. LDB1, Lewa, Leonberg, Germany) used to deliver SC-CO_2_. Pressure in the vessel was measured by a test gauge (mod. MP1, Lecco, Italy) and regulated using a micrometering valve (mod. 1335G4Y, Hoke, Spartanburg, SC, USA). Temperature was regulated using PID controllers (mod. 305, Corsico (MI), Italy). At the exit of the vessel, a rotameter (mod. D6, ASA, Sesto San Giovanni (MI), Italy) was used to measure CO_2_ flow rate.

The vessel was filled with SC-CO_2_; then, when the required pressure and temperature were obtained (200 bar and 35 °C), drying was performed using a SC-CO_2_ flow rate of about 1 kg/h for 5 or 8 h. A depressurization time of about 30 min was used to bring the system back to atmospheric pressure.

### 2.3. Analytical Methods

Field Emission Scanning Electron Microscopy (FESEM) was performed on aerogels previously cryo-fractured using liquid Nitrogen; then, they were sputter coated with Gold (Agar Auto Sputter Coater mod. 108 A, Stansted, UK) at 30 mA for 160 s and analyzed using a FESEM (mod. LEO 1525, Carl Zeiss SMT AG, Oberkochen, Germany) to determine the aerogel morphology and to measure the mean diameter of the nanofibers forming the structure.

Porosity and density measurements were performed on aerogels using an Ultrapycnometer 1000 (Quantachrome instruments, Boynton Beach, FL, USA). Five samples for each process condition were analyzed. Moreover, Brunauer-Emmett-Teller (BET) specific surface area was determined by N_2_ physisorption using an AutoPore IV 9500 V 1.06 by European Micromeritics Analysis Services (Peschiera Borromeo, Milan, Italy). 0.1–0.2 g of aerogel sample was first degassed at 115 °C for 4 h prior to the analysis followed by N_2_ adsorption at −196 °C. BET analysis was carried out at a relative vapor pressure of 0.01–0.3 bar at −196 °C.

Differential Scanning Calorimetry analysis (DSC 30 Mettler, Toledo, Spain) was carried out to identify any change in the thermograms of pure substances compared to A/G aerogels. The analysis was performed in the temperature range between 25 and 400 °C, with a heating rate of 10 °C/min, using Nitrogen as the inert gas.

Aerogels were cryo-fractured using liquid Nitrogen and, then, sputter coated with Chromium (EMITECH K575X peltier cooled); then, they were analyzed by Energy Dispersive X-ray spectroscopy (EDX INCA Energy 350, Oxford Instruments, Gometz la Ville, France) to control the dispersion of the materials in the aerogel matrix; Calcium atoms were selected for alginate and Nitrogen atoms for gelatin.

Free GTA residues released from A/G aerogel were measured in continuous using a Varian (mod. Cary 50) UV/Vis spectrophotometer, reading the absorbance of the sample at 234 nm (the wavelength at which GTA shows maximum absorption) at room temperature.

The aerogel was immersed in a Phosphate Buffer Solution (PBS) at pH = 7.4 and glycine 0.1 M (PBS:Gly = 0.43). We used a solution of PBS at pH = 7.4 to simulate the body environment during GTA release tests from the aerogel. Moreover, we added glycine to the system since PBS tends to precipitate in solid crystals in the release medium and this phenomenon could negatively influence the analysis [20].

Mechanical properties of the aerogels were measured using an INSTRON 4301 (Instron Int. Ltd, High Wycombe, UK). Five rectangular samples for each process condition with a length of 35 mm and a mean thickness of 1.5 mm were specifically prepared following the procedure reported in Section 2.1 and were analyzed using a 100 N load cell, at 1.5 mm/min and 23 °C. All samples were immersed in water for about 20 h before the test. The Young modulus is defined as the initial linear portion of the stress-strain curve. Five specimens were tested for each sample.

## 3. Results and Discussion

### 3.1. Alginate/Gelatin Aerogel Morphology

Scaffold morphology is one of the key characteristics that influences cells adhesion, migration, differentiation, and proliferation [51]. In particular, the organization at nanoscale is required for cell attachment and guidance on the structure; whereas, microporosity is useful to allow the transport of nutrients to the cells [52]. The nanostructure can be naturally introduced in the scaffold if the polymer has the capacity to form a gel. Indeed, in TE application proposals, hydrogels are largely used, due to their similarity to the ECM of the tissue, biocompatibility, and capacity to absorb water [12]. First of all, we analyzed the volume shrinkage of the samples from the stating hydrogel to the final aerogel; all the samples shrank of about 5%–10% during the multistage exchange of the solvent. On the contrary, during supercritical gel drying, which is performed at zero surface tension, the volume remained substantially constant.

**Figure 1:**
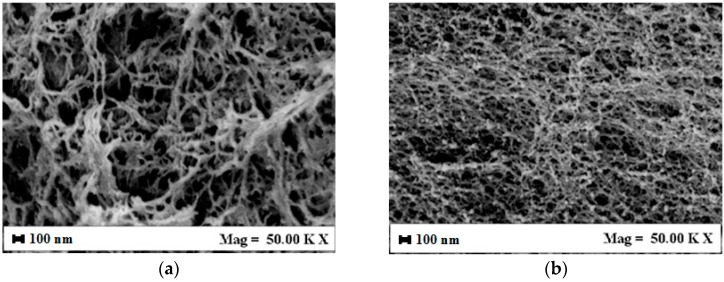
Aerogels morphology after drying at 200 bar, 35 °C for 5 h: (**a**) 5% *w*/*w* gelatin; (**b**) 5% *w*/*w* alginate.

Aerogels formed by A and G alone and their blends, were observed by FESEM to analyze their structure. In Figure 1, pictures of G aerogel and A aerogel at 5% *w*/*w* are reported, for example. G aerogels (Figure 1a) are characterized by a nanofibrous structure, where nanofibers with a mean diameter lower than 100 nm are detectable and form a complex network. Alginate aerogels (Figure 1b) showed a nanoporous homogeneous structure, with a mean pore size of about 100 nm. In both cases, the nanoscale morphology has been preserved, G nanofibers being the most suitable for cell cultivation, since they are more similar to natural ECM [53].

**Figure 2:**
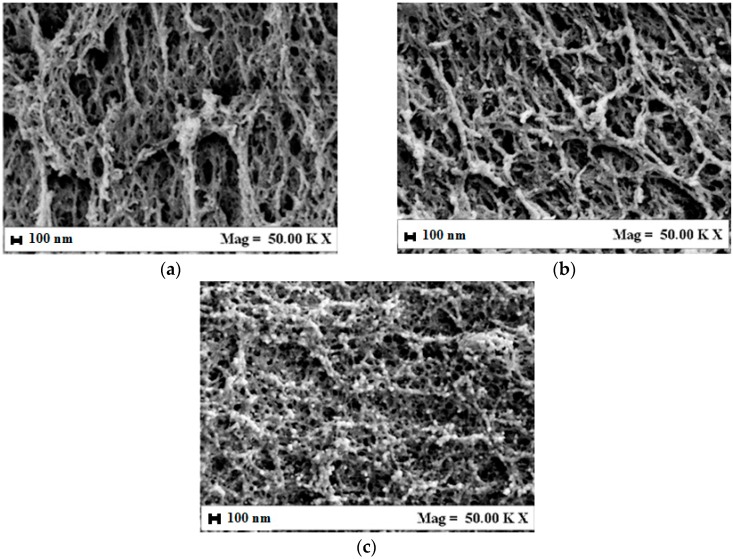
Morphology of A/G blend aerogels: (**a**) A/G 20/80% *v*/*v*; (**b**) A/G 50/50% *v*/*v*; (**c**) A/G 80/20% *v*/*v*.

Then, A/G aerogels morphology was observed. In Figure 2a–c, examples of FESEM images of A/G 20/80% *v*/*v*, 50/50% *v*/*v*, and 80/20% *v*/*v* are shown. The first observation is that A/G aerogels morphology changes with the relative proportion of the two polymers. In particular, it evolves from nanofibrous to nanoporous by increasing alginate percentage in the starting gel, according to the sequence A/G 20/80, 50/50, 80/20. Summarizing, the resulting morphology is similar to that of the polymer contained in the higher percentage and is substantially a hybrid between that of the two polymers when equal percentages are used. As a result, it is possible to select the scaffold structure by changing the polymers’ relative proportions. The supercritical process does not modify gel organization and the avoidance of gel collapse during drying is confirmed also for A/G blends. An explanation of the success of SC-CO_2_ drying is that the operative conditions adopted (*i.e.*, 200 bar, 35 °C) were properly selected to allow the formation of a supercritical mixture (Ethanol + CO_2_), characterized by a negligible surface tension.

Porosity analyses were also performed. We verified that the produced aerogels were highly porous at all polymer compositions tested. In the second column of Table 1, the measured porosity are reported: G aerogel presents a porosity of 95%; whereas A aerogel shows a porosity of 85%. The two-component aerogels present a porosity ranging between about 92% and 88%; *i.e.*, increasing the amount of alginate, the porosity decreases due to the influence of the nanoporous structure that, as expected, is “more compact” that the nanofibrous one. We also analyzed the bulk and skeletal density of the produced aerogels; we found that bulk density varied from 0.016 g/cm^3^ for pure G aerogels, to 0.026 g/cm^3^ for A/G 20/80% *v*/*v* aerogels, to 0.041 g/cm^3^ for A/G 80/20% *v*/*v* aerogels, and to 0.055 g/cm^3^ for A aerogels. The skeletal density varied from 0.315 g/cm^3^ for pure G aerogels, to 0.327 g/cm^3^ for A/G 20/80% *v*/*v* aerogels, to 0.342 g/cm^3^ for A/G 80/20% *v*/*v* aerogels and to 0.366 g/cm^3^ for A aerogels. The aerogels presented similar values of specific surface area ranging between 227 m^2^/g for G aerogel, to 248 m^2^/g for A/G 50/50 *v*/*v* aerogels and to 271 m^2^/g for A aerogels. This last aspect is relevant for potential TE applications: indeed, a high surface area is necessary to allow extensive cell adhesion.

### 3.2. GTA Elimination from Alginate/Gelatin Aerogels

Gelatin crosslinking with GTA is aimed at reducing gelatin solubility and, thus, its fast degradation in an aqueous environment and to improve its mechanical properties [20]. However, GTA is lethal for living cells; indeed, according to the literature, content of about 3 ppm GTA is enough to block cell reproduction [54]. Therefore, to ascertain GTA content in the produced aerogels, we performed GTA release tests by UV/Vis spectrophotometry.

**Figure 3:**
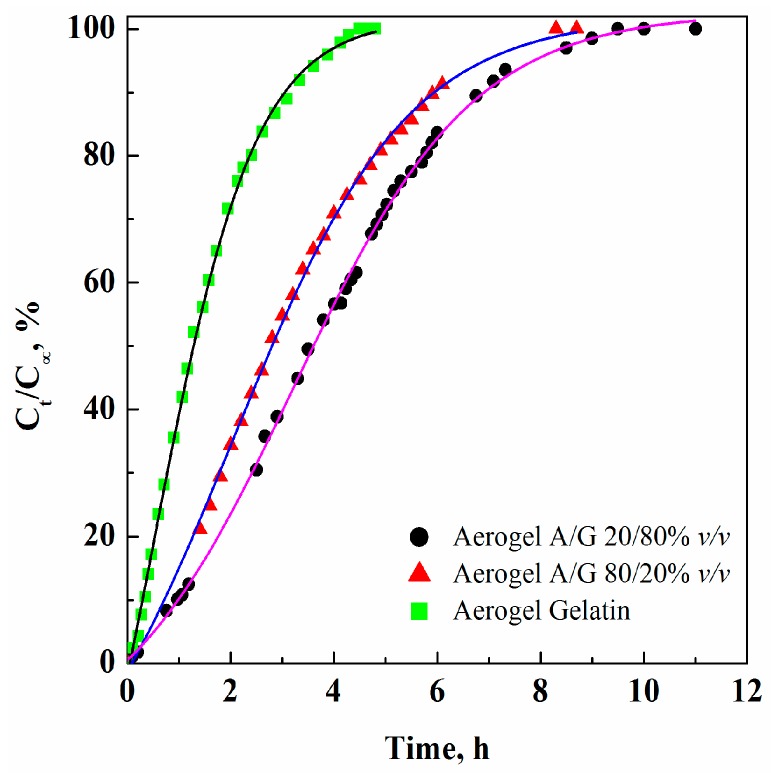
Comparison among GTA release curves from A/G and G aerogels, processed at 200 bar, 35 °C, 5 h.

We performed GTA release analysis reporting the corresponding curves on the same diagram in Figure 3, where normalized GTA concentrations (*i.e.*, GTA concentration released at the time *t*, *C_t_*, divided by the maximum GTA concentration detected for that sample, *C_∞_*) *versus* time are shown. It evidences that the release kinetics depend on the kind of aerogel. We can observe that, in all cases, the slope of the curves, *i.e.* the initial release rate, is different. In particular, to release 50% GTA: 1.2, 2.8 and 3.6 h are required, for the aerogels G, A/G 80/20 and A/G 20/80, respectively. This result depends on both the quantity of free (unreacted) GTA and on the aerogels’ morphology. This last indication can be explained considering that nanofibrous structure is characterized by larger porosities that allow larger mass transfer rates inside the structure during GTA extraction.

After SC-CO_2_ drying for 5 h, the GTA concentration was 21.5 ppm for A/G 80/20 aerogel. In the other samples, GTA concentration decreased when the gelatin amount increased in the aerogel. In the case of G aerogel, GTA residue concentration was about 4 ppm. These GTA concentrations are very small; but in all cases are larger than the GTA level that assures no toxicity for living cells, as previously discussed. To explain these results we have to consider that GTA reacts only with the –NH_2_ groups of lysine and hydroxylysine present in the gelatin structure [20] and GTA solution was added in excess with respect to the stoichiometric ratio; therefore, when gelatin amount was reduced in the polymeric blend, a lower number of NH_2_ groups was involved in the reaction, leaving larger quantities of unreacted GTA.

To force GTA final content to values lower than 3 ppm, we performed longer supercritical gel drying treatments, increasing the drying time from 5 to 8 h. Indeed, it has been shown, in a previous work on GTA elimination from Chitosan aerogels by supercritical gel drying [22], that by increasing the process time, GTA content can be reduced. The results of GTA release tests from the various polymer blends after 8 h drying, are reported in the fourth column of Table 1. Only G and A/G 20/80 aerogels showed GTA levels lower that 3 ppm. In the other cases, longer drying/GTA extraction times are still required.

### 3.3. DSC and EDX Analyses

**Figure 4:**
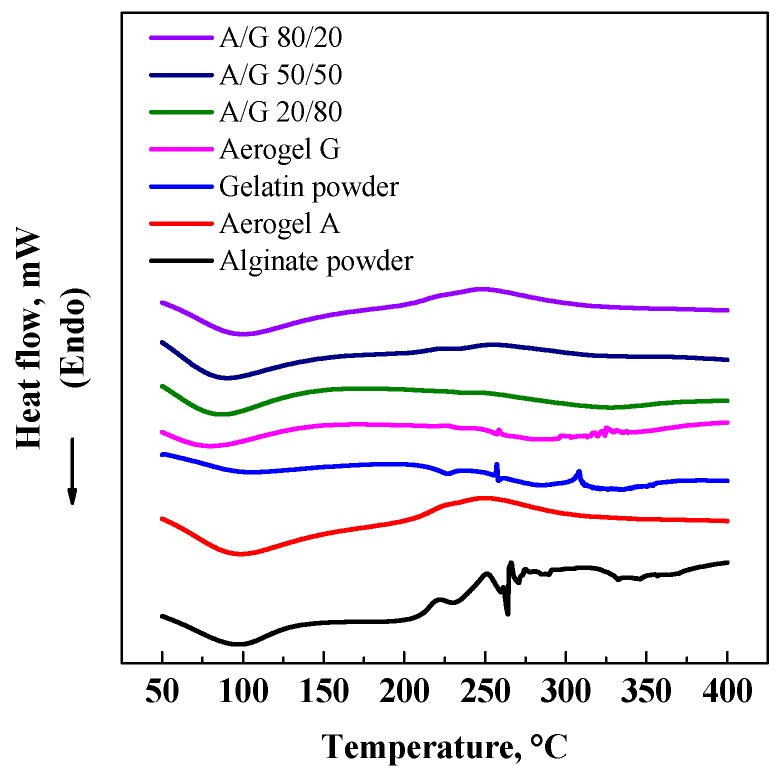
DSC analysis performed on: 80/20% *v*/*v* A/G aerogel; 50/50% *v*/*v* A/G aerogel; 20/80% *v*/*v* A/G aerogel; G aerogel, G powder, A Aerogel, and A powder.

DSC analysis was performed on A and G aerogels and on A/G mixture aerogels to determine the possible changes in the thermal behavior of materials after polymer mixing and processing. Similar thermograms were obtained for all the processed materials (Figure 4), confirming that supercritical processing did not influence the physico-chemical characteristics of the final structures and that the polymers are compatible.

**Figure 5:**
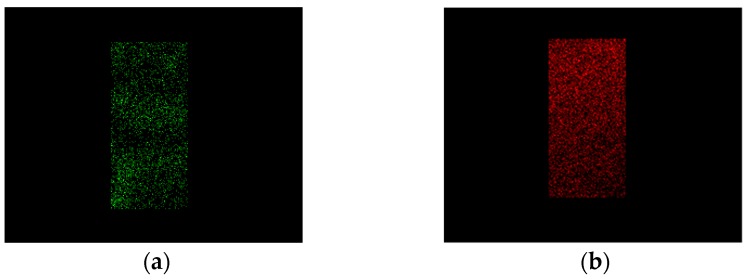
50/50% *v*/*v* A/G aerogel maps: (**a**) Nitrogen atoms, gelatin; (**b**) Calcium atoms, alginate.

We also analyzed the two polymers’ contribution inside the aerogel by EDX, taking advantage of the fact that gelatin presents Nitrogen atoms, indicated in green in the EDX map, and alginate shows characteristic Calcium atoms, that are reported in red in the EDX map. In Figure 5, element maps identifying alginate and gelatin in an A/G aerogel 50/50% *v*/*v* are reported. These images show that G and A are uniformly dispersed in the aerogel: the area covered by Nitrogen overlaps the Calcium area. This interesting result is a consequence of the polymers compatibility, but, also of the fast supercritical process that avoided possible polymer demixing inside the hydrogel matrix during drying. A uniform distribution of the two polymers is required to assure homogeneous biological properties and mechanical behavior of the aerogel.

### 3.4. Mechanical Tests

In the last part of the work, we focused attention on the mechanical characteristics of the A/G aerogels. Tensile mechanical properties were measured and the results obtained are summarized in Table 2.

The results show the effect of A/G composition on the mechanical characteristics of the aerogels produced. The aerogels of single polymers are characterized by a higher Young modulus for gelatin (0.91 MPa) and a higher tensile strength at break for alginate (2.78 MPa). Combining the two polymers, intermediate values and their variation are obtained.

These results confirm that it is possible to continuously modulate A/G aerogels mechanical properties, using the capability of gelatin to increase the elasticity of alginate. The presence of alginate increases the tensile strength of gelatin. Therefore, also at the level of mechanical properties, the integration between the two polymeric gels has been successful.

Moreover, the Young modulus values of the A/G aerogels obtained from the mechanical tests ranged from about 0.6 to 0.9 MPa. These values fall in the range suitable for vascular applications, considering that normal blood vessels are characterized by elastic moduli in the range of 0.2–0.6 MPa under physiological pressures [55].

## 4. Conclusions and Perspectives

The interpenetration of alginate and gelatin hydrogels has been successfully obtained and preserved by SC-CO_2_ drying; it allows modulation of morphology and mechanical properties of these polymer blends. This overall result was made possible by the fact that supercritical drying process allows us to preserve the hydrogels nanostructure in the corresponding aerogels. Moreover, no modifications were found in FESEM and DSC analyses.

In the future, we will study the possibility of adding a porogen to the starting hydrogels with the aim of generating the macroporous structure suitable for cell movement inside the structure. Also biological tests will be performed to analyze the behavior of the structures and of their biological sites and morphology, for potential TE applications.

These results could open the way to the production of improved polymeric scaffolds, taking advantage of the specific characteristics of each polymer used in the IPN.

## Figures and Tables

**Table 1:** Porosity values, GTA concentration detected, bulk and skeletal density, and specific surface area for A/G aerogels and pure A and G aerogels, produced at 200 bar, 35 °C for 5 or 8 h SC-drying.

Aerogel	Porosity (%)	*C*_GTAmax_@5 h (ppm)	*C*_GTAmax_@8 h (ppm)	Bulk density (g/cm^3^)	Skeletal density (g/cm^3^)	Specific surface area (m^2^/g)
G	95.0 ± 3.2	4.2	1.4	0.016	0.315	227
A/G 20/80% *v*/*v*	92.1 ± 3.1	6.6	2.8	0.026	0.327	235
A/G 50/50% *v*/*v*	89.9 ± 2.8	9.5	5.1	0.034	0.335	248
A/G 80/20% *v*/*v*	88.3 ± 2.7	21.5	6.8	0.041	0.342	260
A	84.8 ± 1.9	–	–	0.055	0.366	271

**Table 2:** Comparison among tensile mechanical properties of A/G aerogels and A and G aerogels, processed at 200 bar, 35 °C, 5 h.

Aerogel	Young modulus (MPa)	Tensile strength at break (MPa)
G	0.91 ± 0.11	1.41 ± 0.15
A/G 20/80% *v*/*v*	0.85 ± 0.08	1.92 ± 0.18
A/G 50/50% *v*/*v*	0.78 ± 0.06	2.33 ± 0.25
A/G 80/20% *v*/*v*	0.61 ± 0.05	2.54 ± 0.30
A	0.48 ± 0.03	2.78 ± 0.36
